# Folliculogenesis in random start protocols for oocytes cryopreservation: quantitative and qualitative aspects

**DOI:** 10.1007/s43032-022-00945-8

**Published:** 2022-04-25

**Authors:** Giulia Galati, Nicole Serra, Marta Ciaffaglione, Monica Pinna, Marco Reschini, Valerio Pisaturo, Edgardo Somigliana, Ludovico Muzii, Francesca Filippi

**Affiliations:** 1 Department of Obstetrics and Gynecology, Sapienza University of Rome, Rome, Italy Viale del Policlinico 155, 00161; 2 Department of Clinical Sciences and Community Health, University of Milan, Milan, Italy; 3 Infertility Unit, Fondazione IRCCS Ca’ Granda Ospedale Maggiore Policlinico, Milan, Italy

**Keywords:** Fertility preservation, Oocyte, Random start, Cancer, Ovarian hyperstimulation

## Abstract

Random start protocols are commonly used for oocytes cryopreservation in women with cancer. However, evidence to support their effectiveness is yet modest. This study aims to compare the quality of ovarian response between the ovary carrying the dominant follicle or the corpus luteum (active ovary) and the contralateral ovary (resting ovary). Women with a diagnosis of malignancy who underwent oocytes cryopreservation were reviewed. The main inclusion criterion was the presence of a unilateral dominant follicle or a unilateral corpus luteum on the first day of ovarian hyperstimulation. The primary outcome was the number of mature oocytes retrieved. Intra-patient comparisons between the two ovaries were made using the nonparametric Wilcoxon test for paired data. Forty-three women were included. The number of mature oocytes retrieved from the active and the resting ovaries did not differ, the median [interquartile range—IQR] being 4 [2–7] and 5 [2–8], respectively (*p* = 0.09). The rate [IQR] of mature oocytes per developed follicle was 58% [40–80%] and 65% [33–87%], respectively (*p* = 0.42). In addition, no significant difference emerged when repeating the analyses separately for women carrying dominant follicles and for those carrying corpora lutea. This study failed to detect any detrimental effect of the presence of a dominant follicle or a corpus luteus on the ovarian response to hyperstimulation, thus supporting the validity of random start protocols.

## Introduction

Global cancer incidence in women is estimated at around 9.2 million cases annually [1]. The vast majority affects older women, but about 6% of cases occurs before age 35 [1]. The survival rate of young women affected by cancer is improving owing to advances in antineoplastic therapy, but these treatments can damage the reproductive capacity, leading to delayed or arrested puberty, subfertility, or iatrogenic premature ovarian insufficiency [2–4]. For this reason, performing fertility preservation techniques prior to embarking on oncological treatments is now recommended [5].

Cryopreservation of oocytes is the gold standard for fertility preservation in post-puberal cancer patients [5]. To shorten the duration of ovarian hyperstimulation, “random start” protocols were introduced and are gaining consent worldwide [6, 7]. These regimens are based on the observation that ovarian follicles can respond to hyperstimulation regardless of the phase of the ovarian cycle [8].

Several studies supported the efficacy and feasibility of random start protocols [9–12]. A recent systematic review that included nine comparative studies did not document significant differences between women undergoing a random start protocol and those treated with a conventional hyperstimulation regimen initiated in the early follicular phase. The number of mature oocytes retrieved was similar (weighted mean differences + 0.40 oocytes, 95%CI: -0.84 / + 1.66) [13]. However, none of these studies was randomized and, therefore, the quality of the evidence is not high [13]. Moreover, long-term evidence on the chance of live birth (the most relevant outcome) is lacking because the number of women who have thawed the stored oocytes is yet modest. Investigating more in-depth the effectiveness of random start protocols remains a priority.

In this regard, one may argue that the presence of dominant follicles or corpora lutea could display detrimental effects on the surrounding growing follicles [14]. These functional cysts can secrete several factors with paracrine functions that can potentially interfere with the response to ovarian hyperstimulation. They include sex steroids and their metabolites [15], angiogenic factors [16] and members of the transforming growth factor-beta (TGF-β) superfamily [17]. Previous evidence from our group failed to highlight a detrimental effect on the quantitative aspect of ovarian response, but data on the quality of the response are lacking [14].

In the present study, we aimed at providing additional evidence on the validity of random start protocols by comparing the quality of the ovarian response between the ovary carrying the dominant follicle or the corpus luteum and the contralateral resting ovary. The primary outcome was the rate of mature oocytes that could be retrieved.

## Materials and methods

Women with the diagnosis of malignant tumors who underwent oocytes cryopreservation in the Infertility Unit of Ospedale Maggiore Policlinico in Milan between January 2017 and March 2021 were reviewed. The present study is an extension of a recent study of our group [14]. The main inclusion criterion was the presence of a unilateral dominant follicle (i.e., mean diameter > 11 mm) or a unilateral corpus luteum on the first day of ovarian hyperstimulation. Exclusion criteria were as follows: (1) previous ovarian surgery; (2) presence of ovarian cysts; (3) last menses happened earlier than 5 days before; (4) presence of bilateral dominant follicles or corpora lutea; (5) cycle cancellation stimulation before oocytes retrieval; (6) no previous sexual intercourses because in these cases monitoring was done using transabdominal ultrasound, a technique with insufficient accuracy for our study. We considered only the first ovarian hyperstimulation cycle in those women who had multiple cycles. The local Institutional Review Board (Comitato Etico Area B—Milano) approved our experimental protocol. Only women who gave their consent for their data to be used for retrospective research were included in our study.

All women underwent transvaginal ultrasound for the assessment of antral follicle count (AFC) and possible gynecological disorders prior to initiating the cycle. The presence of a dominant follicle (mean diameter ≥ 11 mm) or a corpus luteum was systematically recorded. A diagnosis of corpus luteum was made in the presence of a unilocular cyst, less than 3 cm in diameter and with diffusely thick-walled and prominent peripheral blood flow (“ring of fire” on Doppler) [18]. Those who accepted to cryopreserve their oocytes started gonadotropins the same day, while those who required more time to take the decision had the possibility to refer some days later. In these cases, the ultrasound was repeated the day of initiation. For ovarian hyperstimulation, a combination of Corrifollitropin, recombinant FSH and GnRH antagonists was administered. Women with hormone-sensitive breast cancer also had letrozole tablets 5 mg given daily. Ovulation trigger with GnRH agonists was given when at least three follicles reached 18 mm. The pre-existing dominant follicle was excluded from this follicle count. That day an in-depth US assessment was performed, and all follicles with a mean diameter ≥ 11 mm were recorded. Oocytes were collected 36 h after GnRH agonists injection. Oocyte denudation, maturation check, and oocyte cryopreservation have been performed 38 h post triggering.

The number of oocytes retrieved as well as the number of mature oocytes was counted separately for the two ovaries. Clinical history was obtained from patients’ charts. Two of the authors with long-lasting experience in gynecological sonography (F.F. and E.S.) performed all the scans.

The primary aim of the study was to compare the number of mature oocytes retrieved between the two ovaries. Secondary outcomes were the rate of oocytes retrieved per developed follicle, the rate of mature oocytes per developed follicle, and the rate of mature oocytes per retrieved oocyte. The ovary with dominant follicle or corpus luteum at the beginning of the hyperstimulation was defined as active, while the other was defined as resting. We stated as clinically relevant demonstrating a lower response in the active ovary in at least two-thirds of cases. Setting type I and II errors at 0.05 and 0.20 and aiming at a 95%CI of ± 15%, this corresponded to about 40 women (http://www.openepi.com). Statistical analyses were performed using the Statistical Package for Social Science (SPSS 23.0, IL, USA). Data were described as mean ± SD, median [Interquartile range: IQR] or number (%), as appropriate. Shapiro–Wilk test was used to verify the normal distribution. The 95% confidence interval (95% CI) of proportions was calculated using a binomial distribution model. Intrapatient comparisons between the two ovaries were made using the non-parametric Wilcoxon test for paired data. P values below 0.05 were considered statistically significant.

## Results

We ultimately enrolled forty-three women, of whom five were also included in our previous study on the quantitative response [14]. Baseline clinical characteristics of the selected women are presented in Table 1. Sixteen (37%) initiated the stimulation in the proliferative phase (presence of a dominant follicle), whereas the remaining twenty-seven (63%) started in the luteal phase (presence of a corpus luteum). The outcome of oocytes cryopreservation cycles is shown in Table 2. The median number of mature oocytes retrieved was 9 [IQR: 6–13].

**Table 1:** Baseline characteristics in the selected women (*n* = 43)

Characteristics	N. (%) or Mean ± SD or Median (IQR)
Age (years)	32 [30—36]
BMI (Kg/m^2^)	21.5 [20.3—24.0]
Smoking	7 (16%)
Previous deliveries	5 (12%)
Seeking pregnancy at the time of cancer diagnosis	5 (12%)
Serum AMH (ng/ml)	2.2 [1.3—3.4]
Total AFC	16 [12 - 21]
Indication to oocytes cryopreservation	
Breast cancer	22 (51%)
Lymphoma	12 (28%)
Other cancers	9 (21%)

N: Number, SD: standard deviation, IQR: interquartile range

**Table 2:** Cycle outcomes in the selected women (*n* = 43)

Characteristics	N. (%) or Mean ± SD or Median [IQR]
Cycle phase at initiation of ovarian hyper-stimulation	
Follicular phase	16 (37%)
Luteal phase	27 (63%)
Total dose of recombinant FSH (IU) ^a^	2,150 [1,925—2,400]
Duration of stimulation (days)	11 [10-12]
N. of women using letrozole	17 (40%)
N. of developed follicles (diameter ≥ 11 mm)	8 [4-11]
N. of oocytes retrieved	12 [7-17]
N. of mature oocytes retrieved (frozen)	9 [6-13]
Rate of oocytes retrieved per developed follicle	78% [60—100%]
Rate of mature oocytes per developed follicle	61% [46—77%]
Rate of mature oocytes per retrieved oocyte	78% [67—100%]

^a^ In women using corrifollitropin, the doses of 100 and 150 ug were considered equivalent to 1,050 and 1,400 IU, respectively

**Fig. 1:**
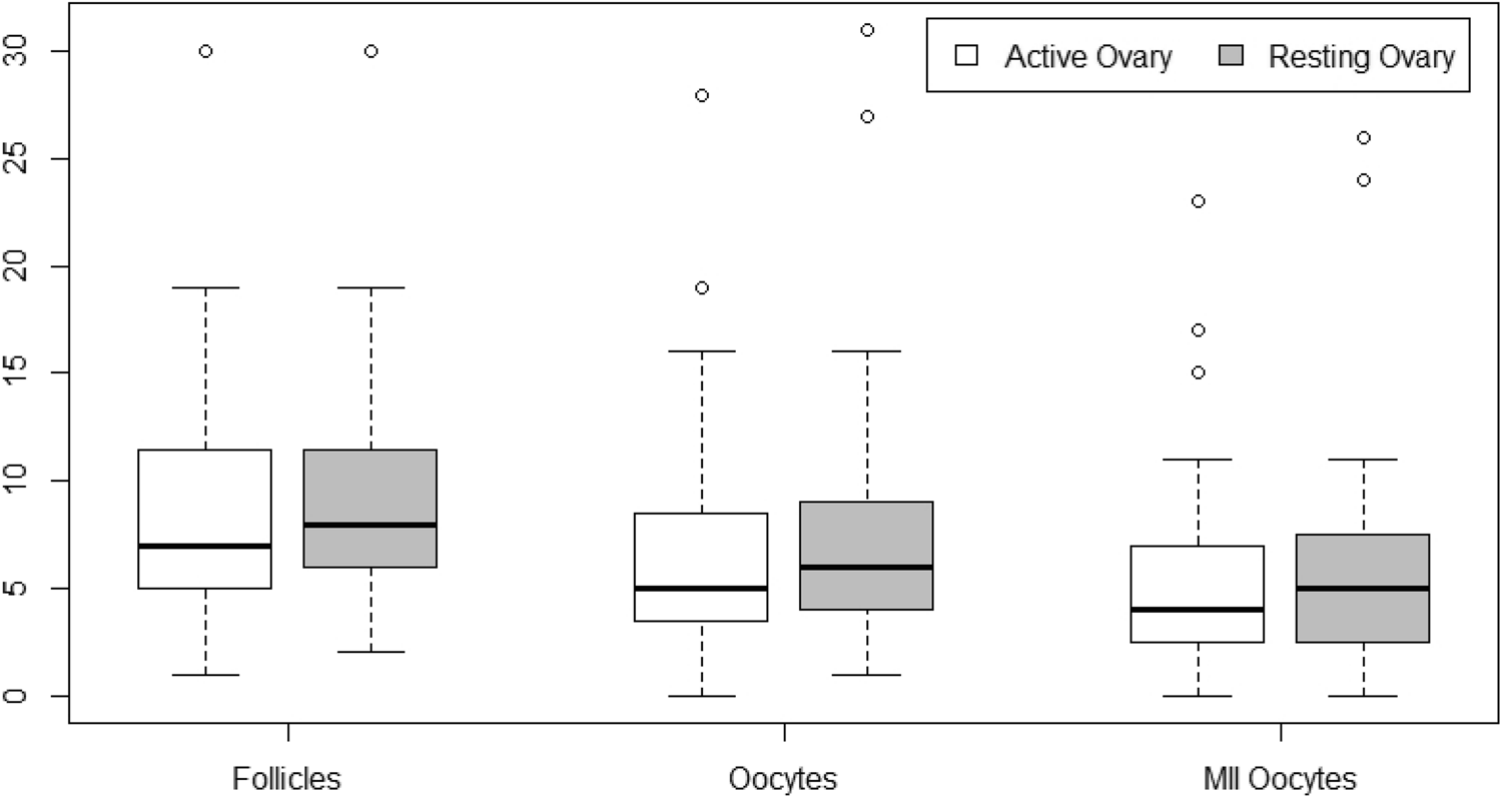
Ovarian response to hyperstimulation in the active ovary (represented in white) and the contralateral resting ovary (represented in gray). The number of developed follicles, the number of oocytes retrieved and the number of mature oocytes (metaphase II oocytes) did not differ

In the active ovary, a lower number of mature oocytes was observed in 26 cases (60%, 95%CI: 46–74%). Moreover, a lower number of developed follicles and oocytes retrieved in the active ovary occurred in 20 cases (47%, 95%CI: 33–61%) and 25 (58%, 95%CI: 43–72%) women, respectively. The direct comparison of these variables also failed to highlight significant differences. The median [IQR] number of mature oocytes retrieved in the active and resting ovaries was 4 [2–7] and 5 [2–8], respectively (*p* = 0.09). The median [IQR] number of developed follicles was 7 [5–12] and 6 [8–12], respectively (*p* = 0.40). The median [IQR] number of oocytes retrieved was 5 [3–9] and 6 [4–9], respectively (*p* = 0.08). These results are illustrated in Fig. 1.

Qualitative analyses aimed at capturing possible detrimental effects on the quality of the folliculogenesis did not show a significant difference. The rate [IQR] of oocytes retrieved per developed follicle in the active and resting ovaries was 71% [53–100%] and 76% [57–100%], respectively (*p* = 0.33). The rate [IQR] of mature oocytes per developed follicle was respectively 58% [40–80%] and 65% [33–87%], respectively (*p* = 0.42). The rate [IQR] of mature oocytes per retrieved oocyte was 83% [72–100%] and 89% [67–100%], respectively (*p* = 0.29).

Table 3 illustrates subgroup analyses according to the phase of the cycle. In none of the two groups and for none of the variables compared, did a difference between the active and the resting ovaries emerge.

**Table 3:** Subgroups analyses according to the phase of the cycle at the beginning of the stimulation

Subgroups	Follicular phase (*n* = 16)			Luteal phase (*n* = 27)		
	Active ovary	Resting ovary	*p*	Active ovary	Resting ovary	*p*
Developed follicles	7 [5-9]	8 [6-12]	0.20	8 [5-12]	8 [5-12]	0.88
Oocytes retrieved	5 [3-8]	6 [3-9]	0.21	5 [3-9]	7 [4-10]	0.20
Mature oocytes retrieved	4 [2-6]	4 [1-7]	0.32	4 [2-7]	6 [3-9]	0.18
Rate of oocytes retrieved per developed follicle (%)	78 [58–100]	67 [52–94]	1.00	70 [47–100]	88 [64–103]	0.15
Rate of mature oocytes per developed follicle (%)	59 [42–87]	58 [33–66]	0.50	58 [40–77]	77 [50–88]	0.13
Rate of mature oocytes per retrieved oocyte (%)	83 [67–100]	90 [50–100]	0.72	81 [74–100]	89 [67–100]	0.22

Data are reported as median [interquartile range] and compared using the paired nonparametric Wilcoxon test

## Discussion

The present study was conducted to assess the effect of dominant follicle or corpus luteum on the quality of ovarian response in women with cancer undergoing random start protocols for oocytes cryopreservation. The results did not show any detrimental effect. Indeed, the number of mature oocytes did not differ between the two ovaries. In addition, the rate of oocytes retrieved per developed follicle, the rate of mature oocytes per developed follicle and the rate of mature oocytes per retrieved oocyte were also similar. These negative findings persisted even when data were analyzed separately according to the phase of the cycle.

Our findings are globally in line with those emerging from the recent systematic review of comparative studies investigating the effectiveness of random start protocols compared to conventional hyperstimulation [13]. This meta-analysis documented a slightly higher need for gonadotropins (2,688 ± 660 versus 2,576 ± 801 IU, *p* = 0.002) but a similar number of mature oocytes retrieved (13.2 ± 3.7 versus 12.6 ± 4.0, *p* = NS). However, the non-randomized design of the included studies hampers firm conclusions. In this regard, our study design is innovative in this area and merits to be underlined. In contrast to comparative studies, the intra-patient comparison of ovarian response between the two gonads of the same woman allows to overcome inter-patient differences and related confounders. This study design should be viewed as highly accurate for the evaluation of the detrimental effects of functional cysts. Both dominant follicles and corpora luteum indeed secrete a plethora of paracrine factors that could theoretically interfere with the normal development of the adjacent follicles [15–17]. Indirectly, this evidence further and differently supports the validity of random start protocols.

Overall, our data support the validity of random start protocols in terms of quality and quantity of ovarian response. More data is however needed on the real competence of these oocytes, i.e., on their capacity to lead to a healthy pregnancy. In this regard, one must also consider possible detrimental effects on uterine function and receptivity. There is for instance emerging evidence that women previously exposed to chemotherapy have a higher prevalence of preterm birth and low birth weight [19]. Unfortunately, evidence on the chances of live birth in cancer survivors with the use of eggs stored at the time of cancer diagnosis is scant. A recent systematic review showed that most studies were poorly informative case reports [20]. Only three case series including 11 [21], 49 [22] and 80 [23] women were identified. The chances of live birth were 15%, 29% and 31%, respectively. However, the small sample size of these case series and the paucity of studies make difficult to draw robust conclusions. To note, data were not presented separately for women who were treated with conventional protocols and for those treated with random start protocols.

Albeit indirect, some reassuring findings were obtained from other areas. Of relevance here is the observation that in dual stimulation cycles (i.e., when a second hyperstimulation is initiated immediately after the first oocytes retrieval) the blastocyst rate for the two cohort of retrieved oocytes was similar [24]. This evidence indirectly supports the validity of random start stimulation in terms of the quality of ovarian response.

Random start protocols have become the standard of care for fertility preservation in women with cancer in the absence of robust evidence [11, 13]. They provide a significant advantage in urgent settings of fertility preservation. They allow to minimize delays to cancer treatment and may create an opportunity to attempt fertility preservation for women who previously did not have the chance owing to time constraints [25]. However, long-term evidence is still needed to definitively corroborate the validity of this approach. In the meantime, the scientific community is called for pursuing means to investigate this topic actively and thoroughly. Our study was designed with this purpose, and the results are reassuring. Larger studies from independent groups using our study design or different type of investigations (preferably RCTs) are, however, still required for a definitive demonstration that quality of the retrieved oocytes is not affected.

Some limitations of our study should be recognized. Firstly, we could not rule out harmful endocrine effects on both ovaries. Both gonads were indeed exposed to the same systemic conditions. Randomized controlled trials are required to properly address this issue. This study design is however difficult to implement in the urgent setting of fertility preservation for cancer [14]. To note, we cannot also exclude some pre-chemotherapy detrimental effects on ovarian function due to the presence of malignant disorders per se. In this regard, one should note that serum AMH was lower than one could expect based on the mean age of the studied population [26]. However, this possible confounder is not expected to impact our conclusions given that these possible negative effects should similarly impact both gonads. Secondly*,* we cannot exclude that the ovarian reserve may be higher in active compared to resting ovaries. This confounder is in our opinion unlikely. The exclusion of women with a history of ovarian surgery or carrying benign ovarian cysts and the observation that baseline AFC was similar between the two ovaries argue against this concern. Thirdly, our sample size was relatively small. We included only 43 women, hampering precise estimates. On the other hand, the design of the study allowed us paired comparisons, thus boosting the power of the statistical analyses.

In conclusion, our findings support the idea that ovarian hyperstimulation for fertility preservation can start regardless of the ovarian cycle phase without compromising oocyte yield and maturity. However, the results of this study should be interpreted by taking into account the limitations and underlining the need for further and more robust studies.

## Data Availability

Not applicable.

## References

[1] Sung H, Ferlay J, Siegel RL, Laversanne M, Soerjomataram I, Jemal A (2021). Global Cancer Statistics 2020: GLOBOCAN Estimates of Incidence and Mortality Worldwide for 36 Cancers in 185 Countries. CA Cancer J Clin.

[2] Hamre H, Kiserud CE, Ruud E, Thorsby PM, Fosså SD (2012). Gonadal function and parenthood 20 years after treatment for childhood lymphoma: a cross-sectional study. Pediatr Blood Cancer.

[3] Nielsen SN, Andersen AN, Schmidt KT, Rechnitzer C, Schmiegelow K, Bentzen JG (2013). A 10-year follow up of reproductive function in women treated for childhood cancer. Reprod Biomed Online.

[4] Kasum M, Šimunić V, Orešković S, Beketić-Orešković L (2014). Fertility preservation with ovarian stimulation protocols prior to cancer treatment. Gynecol Endocrinol.

[5] Oktay K, Harvey BE, Partridge AH, Quinn GP, Reinecke J, Taylor HS (2018). Fertility Preservation in Patients With Cancer: ASCO Clinical Practice Guideline Update. J Clin Oncol.

[6] Cakmak H, Katz A, Cedars MI, Rosen MP (2013). Effective method for emergency fertility preservation: random-start controlled ovarian stimulation. Fertil Steril.

[7] Letourneau JM, Sinha N, Wald K, Harris E, Quinn M, Imbar T (2017). Random start ovarian stimulation for fertility preservation appears unlikely to delay initiation of neoadjuvant chemotherapy for breast cancer. Hum Reprod.

[8] Baerwald AR, Adams GP, Pierson RA (2012). Ovarian antral folliculogenesis during the human menstrual cycle: a review. Hum Reprod Update.

[9] Checa MA, Brassesco M, Sastre M, Gómez M, Herrero J, Marque L (2015). Random-start GnRH antagonist for emergency fertility preservation: a self-controlled trial. Int J Womens Health.

[10] Kim JH, Kim SK, Lee HJ, Lee JR, Jee BC, Suh CS (2015). Efficacy of random-start controlled ovarian stimulation in cancer patients. J Korean Med Sci.

[11] von Wolff M, Capp E, Jauckus J, Strowitzki T, Germeyer A, FertiPROTEKT study group (2016). Timing of ovarian stimulation in patients prior to gonadotoxic therapy: an analysis of 684 stimulations. Eur J Obstet Gynecol Reprod Biol.

[12] Sarais V, Paffoni A, Pagliardini L, Filippi F, Martinelli F, Mangili G (2017). Long-acting recombinant follicle-stimulating hormone in random-start ovarian stimulation protocols for fertility preservation in women with cancer. Acta Obstet Gynecol Scand.

[13] Alexander VM, Martin CE, Schelble AP, Laufer AB, Hardi A, McKenzie LJ (2021). Ovarian stimulation for fertility preservation in women with cancer: A systematic review and meta-analysis comparing random and conventional starts. J Gynecol Obstet Hum Reprod.

[14] Filippi F, Somigliana E, Busnelli A, Guarneri C, Noli S, Restelli L (2020). The presence of dominant follicles and corpora lutea does not perturb response to controlled ovarian stimulation in random start protocols. Sci Rep.

[15] Devoto L, Henríquez S, Kohen P, Strauss JF (2017). The significance of estradiol metabolites in human corpus luteum physiology. Steroids.

[16] Stouffer RL, Bishop CV, Bogan RL, Xu F, Hennebold JD (2013). Endocrine and local control of the primate corpus luteum. Reprod Biol.

[17] Knight PG, Glister C (2006). TGF-beta superfamily members and ovarian follicle development. Reproduction.

[18] Bonde AA, Korngold EK, Foster BR, Fung AW, Sohaey R, Pettersson DR, Guimaraes AR, Coakley FV (2016). Radiological appearances of corpus luteum cysts and their imaging mimics. Abdom Radiol (NY).

[19] Griffiths MJ, Winship AL, Hutt KJ (2020). Do cancer therapies damage the uterus and compromise fertility?. Hum Reprod Update.

[20] Cobo A, García-Velasco JA, Remohí J, Pellicer A (2021). Oocyte vitrification for fertility preservation for both medical and nonmedical reasons. Fertil Steril.

[21] Specchia C, Baggiani A, Immediata V, Ronchetti C, Cesana A, Smeraldi A (2019). Oocyte Cryopreservation in Oncological Patients: Eighteen Years Experience of a Tertiary Care Referral Center. Front Endocrinol (Lausanne).

[22] Diaz-Garcia C, Domingo J, Garcia-Velasco JA, Herraiz S, Mirabet V, Iniesta I (2018). Oocyte vitrification versus ovarian cortex transplantation in fertility preservation for adult women undergoing gonadotoxic treatments: a prospective cohort study. Fertil Steril.

[23] Cobo A, García-Velasco J, Domingo J, Pellicer A, Remohí J (2018). Elective and Onco-fertility preservation: factors related to IVF outcomes. Hum Reprod.

[24] Cimadomo D, Vaiarelli A, Colamaria S, Trabucco E, Alviggi C, Venturella R (2018). Luteal phase anovulatory follicles result in the production of competent oocytes: intra-patient paired case-control study comparing follicular versus luteal phase stimulations in the same ovarian cycle. Hum Reprod.

[25] Cakmak H, Rosen MP (2015). Random-start ovarian stimulation in patients with cancer. Curr Opin Obstet Gynecol.

[26] Du X, Ding T, Zhang H, Zhang C, Ma W, Zhong Y, Qu W, Zheng J, Liu Y, Li Z (2016). Age-Specific Normal Reference Range for Serum Anti-Müllerian Hormone in Healthy Chinese Han Women: A nationwide Population-Based Study. Reprod Sci.

